# Intern doctors’ feedback on teaching methodologies in pharmacology

**DOI:** 10.4103/0976-500X.72359

**Published:** 2010

**Authors:** Jaykaran, Nilesh Chavda, Preeti Yadav, N. D. Kantharia

**Affiliations:** *Department of Pharmacology, Government Medical College, Surat, Gujarat, India*

Sir,

Pharmacology, similar to other branches of medical science, is an everchanging medical subject. It is accepted that reviewing the teaching and evaluation methods by feedback from students and modification of methodologies accordingly is very important for the undergraduate medical teaching.[1,2]

Since quite some time, we have been following the modified teaching (teacher-assisted learning) program, which is also followed at other medical colleges with or without some modifications.[3,4] With a view to improve, we have decided to get feedback for our teaching methodologies and evaluation patterns from intern doctors who have completed their MBBS course from Govt. Medical College, Surat, as they deal with patients, the concepts of pharmacology become more relevant during internship.

Fifty interns were selected randomly by computer-generated random numbers. Individual interns were approached by one of the investigators with a questionnaire that contained 29 questions, each question having 3–9 options. This questionnaire was based on a previous study[5] and validated by a pilot study done on 15 intern doctors.

We observed that most of the interns had some knowledge in the subject of pharmacology before entering the second year (55.2%, 95% CI 39.7%–69.8%); and 21% (95% CI 11%–36.3%) interns had no idea of the subject before entering the second year. When asked their opinion about pharmacology, 39.4% (95% CI 25.6%–55.2%) interns mentioned that it is “useful and interesting,” 31.5% (95% CI 19%–47.4%) believed that pharmacology is “very useful, practically important and interesting,” and 15.7% (95% CI 7%–30%) mentioned that it is “useful but boring.” Most of the interns found Cardiovascular system (CVS) (30.7%, 95% CI 16.5%–49.9%), Autonomic nervous system (ANS) (19.2% 95% CI 8.5%–37.8%), respiratory system (11.5%, 95% CI 4%–28.9%) and chemotherapy (15.3% 95% CI 6%–33%) interesting. 22.2% (95% CI 9 to 45.2%) interns mentioned chemotherapy, 22.2% (95% CI 9%–45.2%) mentioned (Central nervous system) CNS and 16.6% (95% CI 5.8%–39.2%) mentioned CVS pharmacology as the most useful subjects during their internship. Most of the interns (55.5%, 95% CI 37.3%–72.4%) found interactive classes based on strict bilateral communication most interesting. Not a single student found student seminars interesting. Only 11% (95% CI 3%–28%) found whole class lecture interesting. Majority of interns mentioned that case study and treatment discussion (35.7%, 95% CI 20.7%–54.1%) and group discussion (32.1%, 95% CI 17.9%–50.6%) should be added as a part of regular teaching to make it more interesting and useful. As much as 50% (95% CI 32.6%–67.3%) interns mentioned that they preferred studying pharmacology in the second year from text books and self-prepared notes; and 46.4% (95% CI 29.5%–64.1%) interns mentioned that they preferred studying from the text books only. Not a single student mentioned about the teachers’ notes (class notes). As much as 55.5% (95% CI 39.5%–70.4%) mentioned that they were studying pharmacology regularly because of the tests/viva and interactive classes. 16.6% (95% CI 7.8%–31.8%) mentioned that they were studying pharmacology regularly because of interest in the subject. 25.7% (95% CI 14.1%–42%) interns mentioned that they learn pharmacology by understanding the subject. 57.1% (95% CI 40.8%–72%) mentioned that their method of learning pharmacology was a combination of grasping, understanding and mugging the subject. 40% (95% CI 21.8%–61.3%) interns favored the discussion of topics, such as drugs used in special conditions, such as liver, kidney dysfunctions, sexual dysfunctions, emergency drugs and others, in lectures/practicals of pharmacology classes. 49.9% (95% CI 31%–61.6%) interns rated pharmacology teaching above all in the second year subjects. As much as 41.6% (95% CI 27.1%–57.8%) rated pharmacology as one of the few important subjects of MBBS course. Most of the interns mentioned that pharmacology should be taught also in or after III MBBS course; of these, 41.6% (95% CI 27.1%–57.8%) of them mentioned that there should be only few lectures related to newer drugs and recent advancements in therapy. As much as 38.2% (95% CI 23.9% to 54.9%) interns mentioned that as compared with other subjects, pharmacology teachers are “mostly good and few average” and 29.4% (95% CI 16.8%–46.1%) mentioned “all good and knowledgeable.”

The best sequence to study systems in pharmacology suggested by interns is general pharmacology, ANS, CVS, respiratory system, gastrointestinal system, central nervous system, endocrine system, chemotherapy, autocoids and others.

The usefulness of various evaluation methods in preparing the students for university examinations rated by interns in decreasing order were Tutorial, Problem-based learning, Multiple choice questions (MCQ), Terminal exam and Prelims exam. As much as 48.4% (95% CI 32.5%–64.7%) found practicals related to emergency medicine as most interesting part of pharmacology practicals. 73.3% (95% CI 55.5%–85.2%) mentioned that practicals related to emergency medicine is most useful during internship. 89% (95% CI 75.2%–95.7%) interns mentioned that in-depth knowledge of pharmacology is essential to understand clinical therapeutics. 70.2% (95% CI 54.2%–82.5%) interns favored the incorporation of clinical pharmacology in internship training. Of these, 32% pointed that it should be bed side learning. 31.4% (95% CI 18.5%–47.9%) interns mentioned that they wanted to opt for a career in pharmacology. Most of the interns (60%, 95% CI 42.3%–75.4%) mentioned that there is a good scope for pharmacology in industry and research. Some of the qualities mentioned by interns regarding good pharmacology teachers are knowledge of the subject, good communication skills, ability to maintain rapport with the students and ability to make the subject interesting. Important suggestions regarding the modification in pharmacology teaching curricula are making the subject more clinically oriented, having more interactive classes, more problem-based learning and more MCQs.

In this study, many interns mentioned about not having any knowledge of pharmacology before entering the second year MBBS course. An overview of different subjects at the start of the first year of MBBS course may be helpful. Bilateral communication enhances the students’ involvement in lectures, which might improve their performance in the exams, hence it was the most preferred.[5] Similar to a previous study, student seminar was not popular.[6] Many interns favored the introduction of bed side training of clinical pharmacology during internship, which is also supported by other studies.[7,8] About one third of interns wanted to opt for a career in pharmacology and this shows the growing popularity of pharmacology and the demand for pharmacologists in the industry and research institutes.
